# Vessel wall imaging features of Moyamoya disease in a North American population: patterns of negative remodelling, contrast enhancement, wall thickening, and stenosis

**DOI:** 10.1186/s12880-022-00930-2

**Published:** 2022-11-17

**Authors:** Anthony S. Larson, James P. Klaas, Matthew P. Johnson, John C. Benson, Darya Shlapak, Giuseppe Lanzino, Luis E. Savastano, Vance T. Lehman

**Affiliations:** 1 University of Minnesota Medical School, Minneapolis, MN USA; 2 Department of Neurology, Mayo Clinic, Rochester, MN USA; 3 Department of Quantitative Health Sciences, Mayo Clinic, Rochester, MN USA; 4 Department of Radiology, Mayo Clinic, 200 First Street SW, Rochester, MN 55905 USA; 5 Neurologic Surgery, Mayo Clinic, Rochester, MN USA

**Keywords:** Moyamoya disease, Vessel wall imaging, Negative remodeling, Vessel wall enhancement, Moyamoya, Moyamoya syndrome

## Abstract

**Background:**

This study characterized vessel wall imaging (VWI) features of Moyamoya disease (MMD) in a predominantly adult population at a North American center.

**Methods:**

Consecutive patients with VWI were included. Twelve arterial segments were analyzed for wall thickening, degree and pattern of contrast enhancement, and remodeling.

**Results:**

Overall, 286 segments were evaluated in 24 patients (mean age = 36.0 years [range = 1–58]). Of 172 affected segments, 163 (95%) demonstrated negative remodeling. Complete vessel wall obliteration was most frequent in the proximal M1 (17/48, 35%). Affected segments enhanced in 72/172 (42%) (n = 15 for grade II; n = 54 for concentric and n = 18 for eccentric); 20 of 24 (83%) patients had at least one enhancing segment. Both enhancing and non-enhancing segments were present in 19/20 (95%) patients. Vessel wall enhancement was most common in the proximal segments and correlated to the degree of stenosis (*p* < 0.001), and outer wall diameter (*p* < 0.001), but not disease duration (*p* = 0.922) or Suzuki score (*p* = 0.477). Wall thickening was present in 82/172 (48%) affected segments and was associated with contrast enhancement (*p* < 0.001), degree of stenosis (*p* < 0.001), and smaller outer wall diameter (*p* = 0.004).

**Conclusion:**

This study presents VWI findings in North American patients with MMD. Negative remodeling was the most common finding. Most patients had both enhancing and non-enhancing abnormal segments. Vessel wall enhancement was most common in proximal segments, variable in pattern or degree and was correlated to the degree of stenosis and smaller outer wall diameter.

## Background

Moyamoya disease (MMD) is an idiopathic steno-occlusive disease characterized by progressive stenosis and/or occlusion of the internal carotid artery (ICA) terminus and adjacent M1 and A1 segments. This stenosis or occlusion can be associated with basal collateral vessel formation (lenticulostriate and thalamostriate collaterals), giving the characteristic “puff of smoke” appearance on cerebral angiography. The pathophysiology of MMD has not been completely elucidated, however both genetic and environmental factors are thought to have substantial influence given the high genetic, and potentially phenotypic, variability between populations, ethnicities, and monozygotic twins [1]. For example, the RNF213 protein has been associated with some cases of MMD in the Asian population while recent evidence has identified DIAPH1 mutations has been associated with MMD in the non-East Asian population [2]. It remains uncertain if such genetic differences and/or environmental factors result in an identical or variable imaging phenotype. In general, the prevalence of MMD is much greater in Eastern countries compared to those in North America [1].

One imaging method to evaluate MMD is vessel wall imaging (VWI). A recent systematic review found that intracranial VWI studies to date have been most frequently (> 50%) published from groups in Asia, with fewer originating from North American institutions [3]. Additionally, those focused primarily on MMD comprise a small percentage of publications [3]. Accordingly, there are relatively few studies characterizing the VWI features of MMD in a North American population.

Further, prior studies report highly variable prevalence of VWI findings in MMD; in particular the prevalence and importance of vessel wall enhancement [4]. A lack of characterization could lead to confusion during interpretation of a VWI examination, which may be performed to help distinguish MMD from Moyamoya syndrome (MMS) due to vasculitis/inflammatory etiology or atherosclerosis. Characterization of VWI features is also important to help clinicians start to understand which findings might be the best candidates to assess for potential biomarkers of disease activity. This study further characterized VWI findings of idiopathic MMD evaluated at a North American institution.

## Methods

This was a single center retrospective descriptive study. Institutional review board (Mayo Clinic Institutional Review Board) approval was obtained prior to the initiation of this study. All patients that were included in this study provided written informed consent for general involvement in retrospective research activities at our institution and the Mayo Clinic Institutional Review Board waived the need for additional consent.

### Patient selection and diagnostic criteria of Moyamoya disease

Consecutive patients who presented to our institution and who underwent VWI MRI studies of the intracranial vasculature were retrospectively reviewed. Patients who were found to have intracranial arterial stenosis of the intracranial ICA or M1 segment of the ICA were initially included. The medical records and radiographic studies of each patient initially included were scrutinized to determine if diagnostic criteria for idiopathic MMD was met. The *Guidelines for Diagnosis and Treatment of Moyamoya Disease* (2012) were used as diagnostic criteria [5]. In brief, the diagnosis requires idiopathic stenosis of the bilateral terminal ICAs, proximal M1 segment or proximal A1 segments with an abnormal network of vessels in the basal ganglia. Currently, either DSA or MRA may be used for this diagnosis. Patients with unilateral involvement were excluded as this meets diagnostic criteria for only probable or quasi-MMD, and not definitive idiopathic MMD. Patients found to have an underlying condition that would classify the diagnosis as MMS were also excluded. Such conditions consisted of autoimmune disease, connective tissue diseases, hematologic disorders, prior cranial irradiation, intracranial neoplasm, documentation of prior intracranial infections, or genetic syndrome associated with MMS. Patients with imaging that suggested the presence of intracranial atherosclerosis, dissection or chronic occlusion were excluded.

A board-certified neurologist with vascular expertise performed a chart/imaging review and a board-certified radiologist with a certificate of added qualification in neuroradiology corroborated the imaging features of diagnosis. Importantly, imaging features of HR-VWI were not used for diagnosis since these are not included in these current strict established criteria and to avoid the potential confounder of using the modality being assessed for initial diagnosis.

### Baseline demographic and clinical data

From the medical record of each included patient, we collected demographic data, including age, sex, and ethnicity. Clinical data that was collected included a history of vascular comorbidities as well as the initial presentation of MMD which was categorized as ischemic, hemorrhagic, or other. Ischemic presentations were defined as an ischemic stroke demonstrated on MRI or CT imaging studies or a transient ischemic attack (TIA) as diagnosed by a neurologist. Hemorrhagic presentations were classified as intracranial hemorrhage diagnosed by CT studies. The time from the initial diagnosis of MMD to the completion of HR-VWI studies was also determined. Surgical treatment status at the time of VWI and Suzuki scores were documented.

### Vessel wall imaging protocol

VWI examinations were performed as a 3D SPACE (sampling perfection with application optimized contrasts using different flip angle evolution) at 3 T on a Siemens (Munich, Germany) PRISMA or SKYRA unit with the following parameters: 64 channel head coil, 160 mm × 160 mm field of view, 320 × 320 acquisition matrix, TR 1760 ms, TE 31 ms, echo train length 58, Nex 2, variable flip angle, 0.25 mm × 0.25 mm interpolated matrix size, 0.5 mm slice thickness 0.5, and 6 cm slab thickness. Sequences were performed with proton density weighting without and with IV 0.1 mmol/kg Gadavist (gadobutrol 1 mmol/Kg, Bayer Schering Pharma, Berlin, Germany).

### Vessel wall imaging analysis

All VWI examinations were evaluated by two of three fellowship-trained neuroradiologists and discrepancies were resolved by the third who selected one of the initial scores to arrive at consensus for all features except measurements (initials anonymized). For simplicity, all measurements were performed by a single neuroradiologist (anonymized). Twelve segments were assessed in each patient: the distal supraclinoid ICA, proximal 1/3 M1, mid 1/3 M1, distal 1/3 M1, proximal 1/2 A1, and distal 1/2 A1. Since the length of these segments is variable, each partitioned into equal segments by visual inspection, respectively. Each segment was assessed for outer diameter measurement, lumen measurement, wall thickening, and wall enhancement. The predominant patterns within each segment were recorded. Each segment was viewed using the multiplanar reformat function in Visage PACS (Visage Imaging, Inc., San Diego, CA) to create a double oblique image of the true cross-sectional depiction. Each segment was viewed along the entire length in both axial and cross-referenced double oblique images to identify the single most severely stenotic level by visual inspection. The outer diameter of the outer vessel wall and the lumen were meticulously measured using a full-width half maximum technique to determine boundaries of the outer wall or luminal surface on the double oblique true cross-sectional image in each segment at the most severely affected level as described previously in the literature [6]. In addition to the measurement, the presence or absence of subjective negative remodeling of the outer diameter was determined for each segment (dichotomous yes/no designation); the reason for this method is that it likely mirrors actual clinical practice and, in some cases, there were no normal adjacent segments to establish a quantitative denominator for comparison. Additionally, the remodeling index (RI) of the supraclinoid ICA segments were determined as the ratio of the diameter of the outer wall of the distal supraclinoid ICA to that of the proximal supraclinoid ICA (just past the level of the ophthalmic artery origin). Complete obliteration of a vessel wall segment was defined as a vessel wall that was not identifiable or too small to measure, even if a minimal thin streak of enhancement was present.

Vessel wall thickening was assessed subjectively as absent, concentric, or eccentric relative to adjacent normal vessels. Eccentric wall thickening was defined as thickening that spared at least one portion of the vessel wall in cross-section or thickening that involved the entire circumference but was heterogeneous with one area that exceeded twice the thickness of another. Concentric vessel wall thickening involved the entire cross-sectional circumference of the vessel wall throughout the involved segment and did not meet criteria for eccentric wall thickening (uniform thickening). Vessel segments that had both areas of concentric and eccentric thickening were considered eccentric.

Vessel wall enhancement was characterized by both grade and pattern. Enhancement was graded as either absent, low grade (less intense than the pituitary stalk), or high grade (enhancement equal or greater than the pituitary stalk). Additionally, enhancement was characterized as either concentric or eccentric. Eccentric vessel wall enhancement was defined as enhancement that spared at least one portion of the vessel wall in cross-section or thickening that involved the entire circumference but was heterogeneous with one area that exceeded twice the thickness of another. Concentric vessel wall enhancement involved the entire cross-sectional circumference of the vessel wall throughout the involved segment and did not meet criteria for eccentric wall enhancement (uniform enhancement). Vessel segments that had both areas of concentric and eccentric enhancement were considered eccentric.

### Statistical analysis

Means and ranges were calculated for continuous variables including the age at MMD diagnosis and the time from MMD diagnosis to VWI. Means and standard deviation (SD) were calculated for remodeling indices. Percentages were calculated for categorical variables including sex, ethnicity, vascular comorbidities, presentation and VWI features of each segment. The number and percentage of vessel wall segments demonstrating each of the changes (contrast enhancement, thickening, remodeling) were calculated for each arterial location, as well as for all segments overall. For each of these vessel wall findings, the number and percentage of patients with at least one segment demonstrating each change was calculated. The number and percentage patients demonstrating unilateral and bilateral changes in each of the arterial segments were calculated.

Correlation of enhancement and thickness by segment were evaluated in two ways. First, both enhancement and thickness were dichotomized to present or absent and tested for an association overall and by individual vessels using mixed effects logistic regression to account for clustering by patient. Second, enhancement and thickening were tested for an association using their degrees with mixed effects multinomial regression.

Correlation of enhancement or thickness to vessel outer diameter by segment was completed using mixed effects logistic regression. For this analysis, contrast enhancement was considered either present or absent as a binary state regardless of degree or pattern (any enhancement was considered positive). Similarly, vessel wall thickening was considered either present or absent as a binary state.

Inter-reader agreement was evaluated using the kappa statistic. All findings used their previously calculated binary state (present or absent). Both time from diagnosis and modified Suzuki score were evaluated for association with number of enhancing segments using mixed effects linear regression.

All statistical analysis was completed using R version 3.6.2 (R Foundation for Statistical Computing, Vienna, Austria).

## Results

### Patients, baseline information and arterial segments

Thirty-two patients suspected of having idiopathic MMD who also had VWI performed at our institution were reviewed. Eight patients were excluded from analysis based on the aforementioned criteria; seven met criteria for MMS, and one patient had strictly unilateral stenosis. Accordingly, twenty-four patients diagnosed with idiopathic MMD, who also had HR-VWI studies performed at our institution, were included in the final cohort. The mean age at diagnosis of MMD was 36.0 years (range = 1–58), and 17 (70.8%) were female. None of the patients had undergone surgical bypass (direct or indirect) at the time of the VWI examination. Patient demographics, presentations, and baseline information is further summarized in Table 1. In total, there were 286 intracranial anterior circulation segments that were analyzed from 24 patients (each patient had 12 total segments, the A1 segment was considered congenitally absent on one side in one patient). Thus, there were a total of 48 vessel wall segments (left or right) that were analyzed for each segment, except 47 for both the proximal and distal A1 segments. Suzuki scores were available 21 patients, for the remaining 3 the Suzuki score was determined from MRA data (see raw data).

**Table 1:** Patient demographics and clinical features

Total patients included	24
*Demographics*	
Mean age at Moyamoya diagnosis (range)	36.0 (1–58)
Female, no. (%)	17 (70.8)
*Ethnicity, no. (%)*	
Caucasian	18 (75.0)
Asian	2 (8.3)
African American	2 (8.3)
Other	2 (8.3)
*Vascular comorbidities, no. (%)*	
Hypertension	9 (37.5)
Hyperlipidemia	11 (45.8)
Diabetes mellitus	4 (16.7)
Coronary artery disease	0 (0.0)
Ever smoker	9 (37.5)
*Presentation (%)*	
Ischemia	22 (91.7)
Hemorrhage	1 (4.2)
Other	1 (4.2)
Mean time from diagnosis to VWI in mo. (range)	23.4 (0–202)

### Overall frequency and pattern of affected segments

**Fig. 1:**
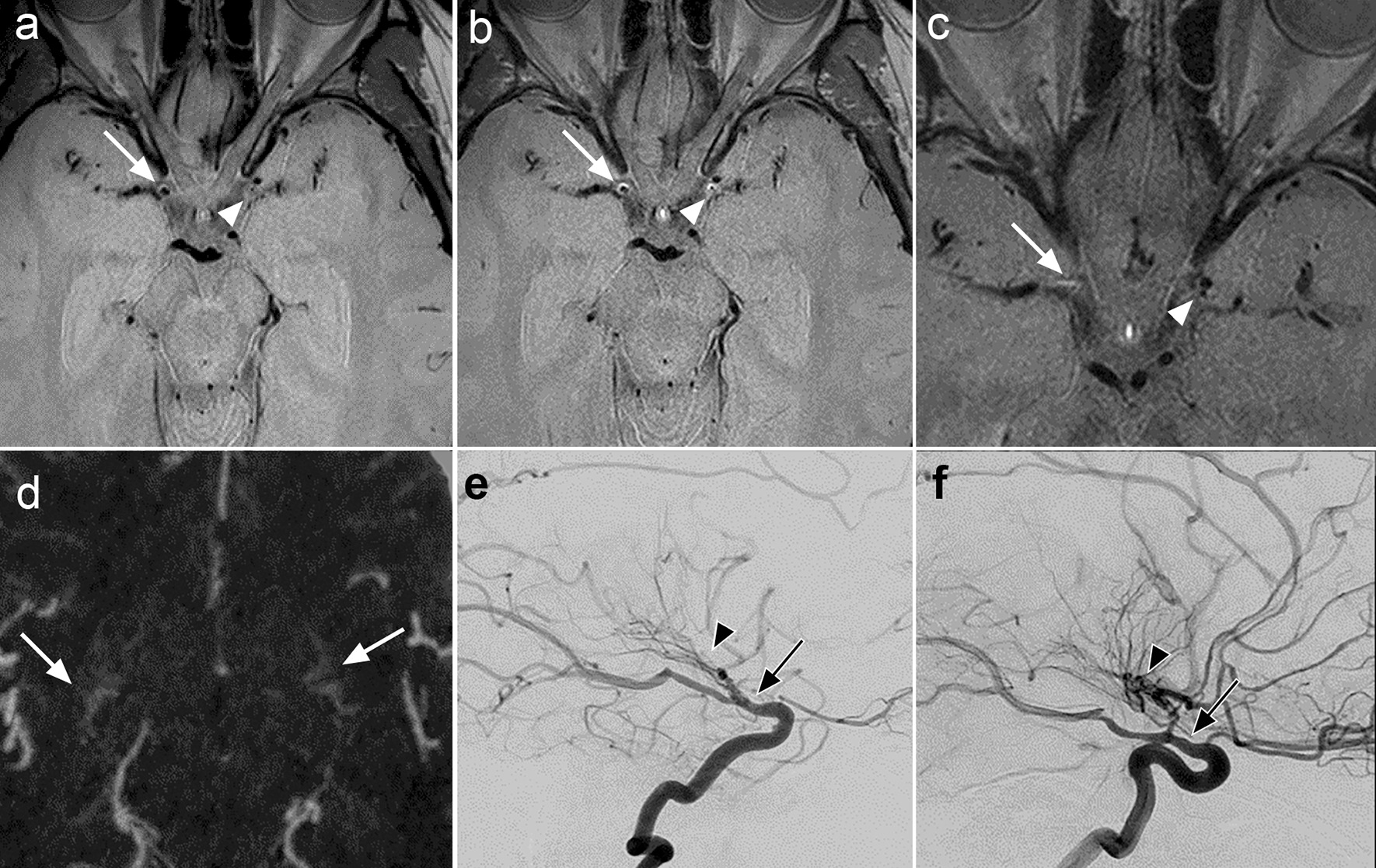
36-year-old female presented with right-sided weakness and border zone infarcts in the left cerebral hemisphere. The patient had a history of borderline hypertension and borderline hyperlipidemia. No other vascular risk factors. No oral contraceptives. No findings of inflammation on CSF testing. Panel to assess for hypercoagulability was negative. The best fit diagnosis was MMD. VWI without gadolinium demonstrates eccentric wall thickening of the distal right ICA (white arrow) and concentric thickening of the distal left ICA (white arrowhead) as well as negative remodeling of the involved segments (**a**). VWI with gadolinium demonstrates eccentric grade I enhancement of the distal right ICA corresponding to the region of thickening (white arrow) and concentric grade I enhancement (the predominant pattern was considered concentric throughout the involved area, though not uniformly concentric on the provided image) of the distal left ICA (white arrowhead) (**b**). The enhancement, in this case on both sides, is intermediate and approaches grade II, but was considered slightly less than the pituitary stalk and designated grade I. A one-year follow-up VWI exam shown for descriptive purposes shows bilateral progressive negative remodeling of the terminal ICAs, proximal M1 segments, and A1 segments without non-contiguous areas of vessel wall abnormality (not shown) as well as decreased vessel wall enhancement (**c**). An axial CTA MIP shows proliferation of bilateral lenticulostriate collateral arteries (white arrows) (**d**). One-year follow-up lateral DSA images on the right (**e**) and left (**f**) shows bilateral smooth tapered stenosis of the terminal ICAs with occlusion of the proximal middle and anterior cerebral arteries and proliferation of bilateral lenticulostriate collateral arteries, more marked on the left. No other steno-occlusive lesions were present in the cervical or intracranial arteries (not shown)

**Fig. 2:**
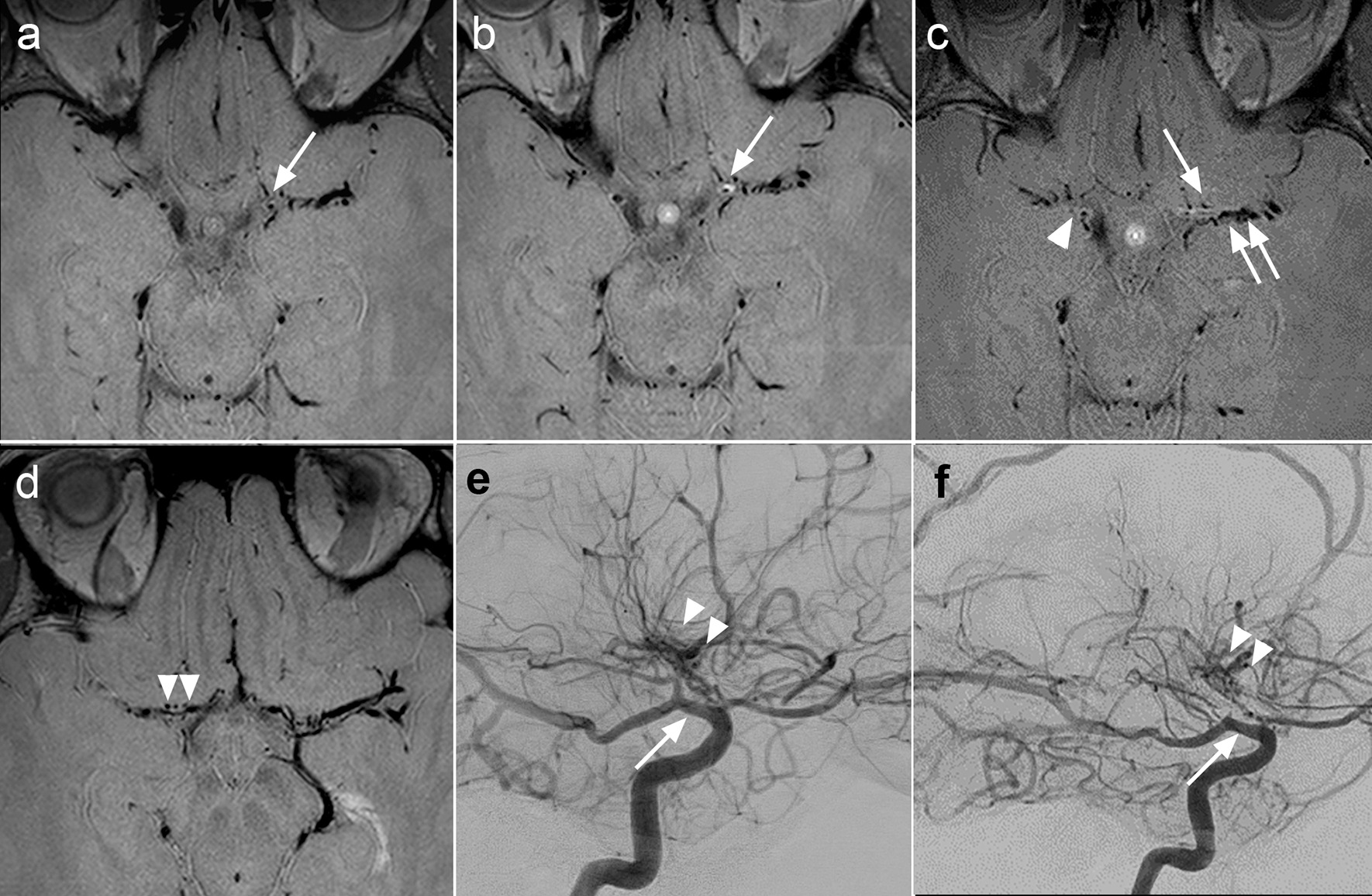
Eccentric grade II enhancement and absent enhancement of more distal involved segments. 31-year-old female with a history of bilateral deep border zone infarcts. The patient had a history of half-pack per day smoking for several years, but no other vascular risk factors. Trans-esophageal echocardiogram results were within normal limits. A comprehensive rheumatologic work-up did not reveal evidence of vasculitis or autoimmune disorder. CSF studies were unremarkable for evidence of vasculitis or active infection. Axial VWI image without gadolinium (**a**) demonstrates subtle eccentric thickening of the anterior wall of the left ICA terminus with negative remodeling (white arrow). Axial VWI with gadolinium (**b**) demonstrates eccentric grade II enhancement of the left ICA terminus corresponding to the area of eccentric thickening (white arrow). Axial VWI images with gadolinium at the level of the M1 segments (**c**, **d**) demonstrates faint (grade 1) circumferential wall enhancement of the distal right ICA (white arrowhead, **c**) proximal right M1 segment (white arrowheads, **d**), and proximal left M1 segment (white arrow, **c**). In distinction, vessel wall enhancement was absent in the mid to distal left M1 segment (double white arrows, **c**). The left middle cerebral vein is seen (double white arrowheads, **f**). Two-year follow-up right lateral (**e**) and left lateral (**f**) DSA images demonstrate progressive marked tapered stenosis of the bilateral ICA termini (white arrows), bilateral proximal A1 segments, and bilateral M1 segments as well as proliferation of lenticulostriate collaterals (white arrowheads), consistent with MMD

**Fig. 3:**
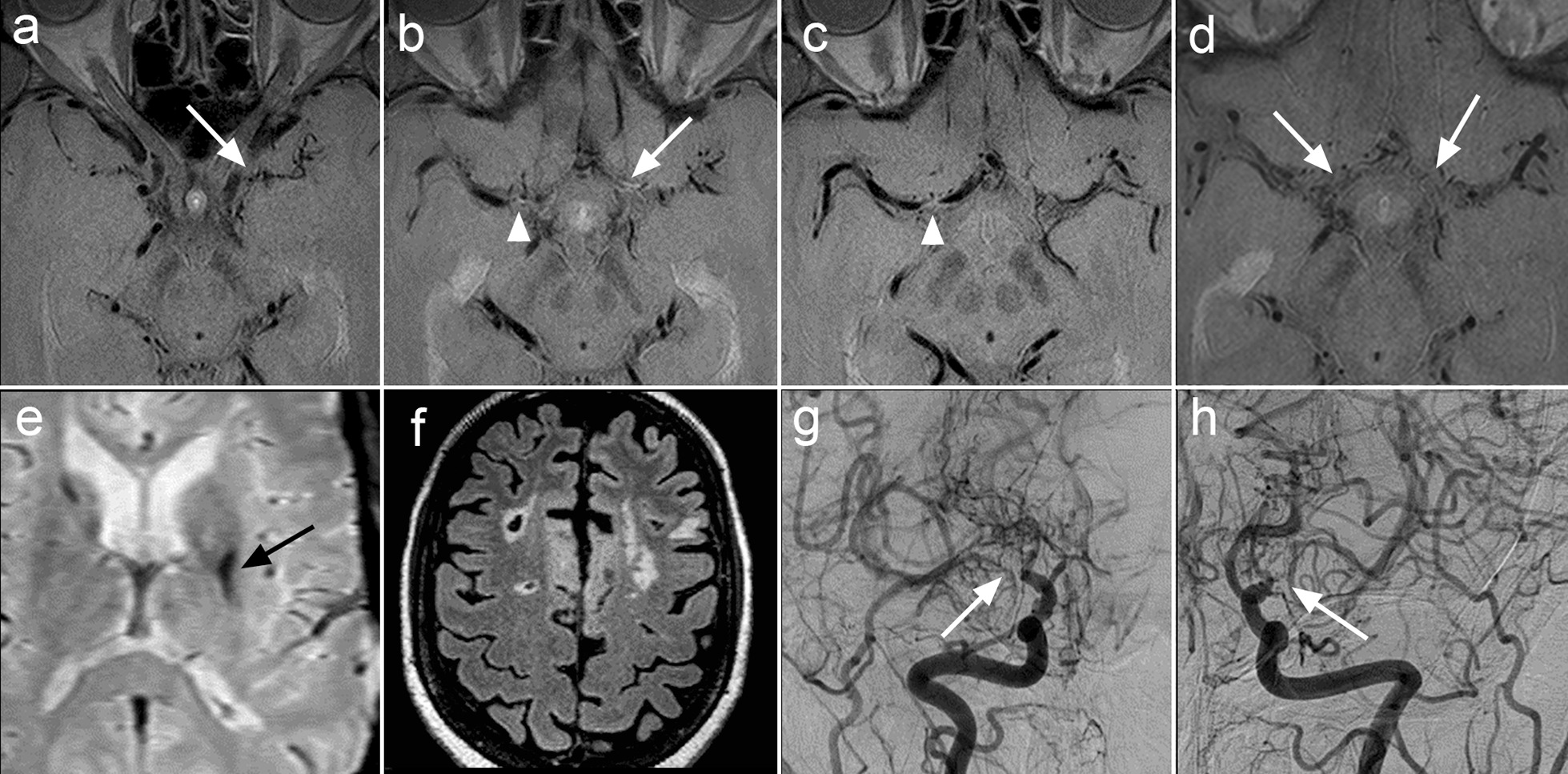
Variable pattern of vessel wall enhancement among different affected segments in a single patient. A 47-year-old female with a 1-year history of recurrent infarcts in the bilateral MCA territories had a history remarkable for a heterozygous factor V Leiden mutation. Otherwise, the patient had no laboratory findings of hypercoagulability (including protein C, protein S, antithrombin III, antiphospholipid antibody). No vascular risk factors, such as smoking, hyperlipidemia/hypercholesterolemia, hypertension, or diabetes. A trans-esophageal echocardiogram was unremarkable. Rheumatologic history and laboratory work-up were also unremarkable with no evidence of vasculitis or autoimmune disorder. Axial VWI demonstrates narrowing and negative remodeling of the supraclinoid left ICA without vessel wall enhancement (white arrow, **a**), while there was grade I circumferential wall enhancement of the distal left ICA (not shown). There was also eccentric grade I enhancement of the posterior wall of the distal right ICA (white arrowhead) as well as grade I circumferential wall enhancement of the left A1 segment at the same level (white arrow, **b**). The left M1 segment demonstrates negative remodeling throughout without vessel wall enhancement (white arrow, **c**). There is marked focal negative remodeling at the junction of the right M1 and A1 segments with faint grade I enhancement (white arrowhead) and sparing of the mid to distal right M1 segment and distal right A1 segment (**c**). Two-year follow-up VWI for descriptive purposes showed that there were no identifiable remnants of the terminal ICAs, proximal M1 or proximal A1 segments (white arrows) (**d**). An axial gradient echo image shows chronic blood product in the left lentiform nucleus (black arrow, **e**). An axial T2 FLAIR image shows chronic infarcts of the bilateral cerebral hemispheres (**f**). Right frontal (**g**) and left frontal (**h**) DSA images (CCA injections) further show the bilateral areas of stenosis and proliferation of lenticulostriate vessels, more marked on the right

Overall, 286 segments were evaluated in 24 patients. Two segments were not evaluated due to a single congenitally absent A1 artery (absent proximal and corresponding distal A1 segments). One hundred seventy-two of 286 (60%) segments were affected as defined by any abnormality of the vessel wall (remodeling, any pattern or degree of enhancement, and/or any pattern of thickening). The distal ICA (36/48, 75%), proximal M1 (33/48 69%), and proximal A1 segments (33/47 70%) were the most commonly affected segments. The mid M1 segment was affected in 28/48 (58%) cases, the distal M1 in 24/48 (50%) cases, and the distal A1 in 20/47 (43%) instances. Examples of patterns of vessel wall findings, which were most marked proximally, with varying patterns of contrast enhancement, remodeling, and thickening as detailed below are presented in Figs. 1, 2 and 3.

### Vessel wall remodeling and luminal measurements

**Fig. 4:**
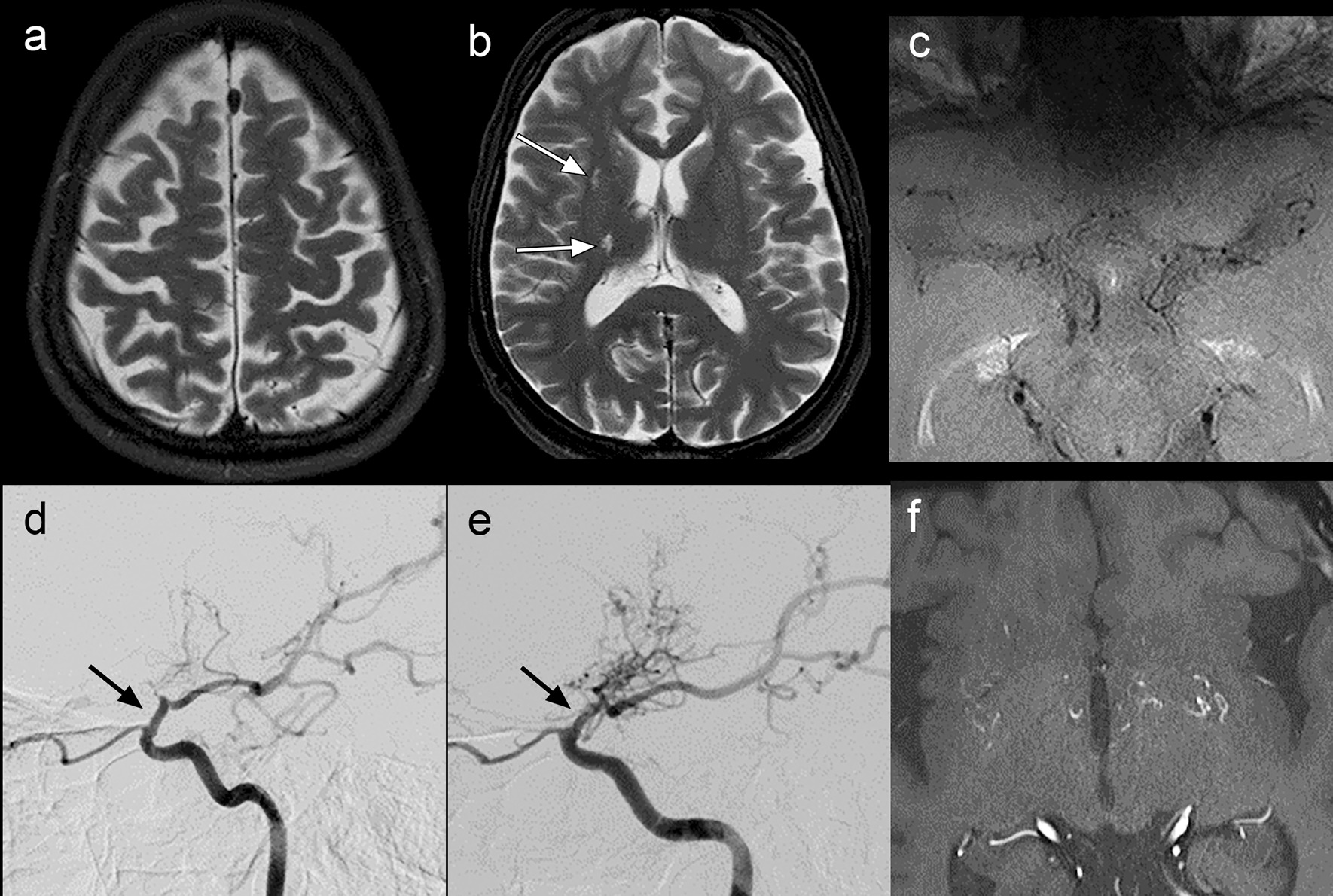
Example of a completely obliterated arterial appearance on VWI. A 44-year-old female with a 2-year history of paresthesia and clinical findings compatible with right hemispheric infarcts had no vascular risk factors, an unremarkable work-up for rheumatologic conditions or other causes of a Moyamoya syndrome. MRI head, including axial FSE T2 weighted images (**a**, **b**) demonstrate greater than expected generalized volume loss for age in the bilateral cerebral hemispheres and chronic lacunar infarcts (white arrow, b) in the right internal capsule, subinsular white matter (white arrow, **b**), coronal radiata (not shown), and caudate head (not shown). Axial VWI with contrast (**c**) demonstrates complete non-visualization of M1 segment arteries in the expected MCA cistern regions (on this and all other adjacent images), compatible with a VWI appearance of complete obliteration. Right lateral DSA (**d**) and left lateral DSA (**e**) show expected tapering of the terminal ICAs bilaterally (black arrows) and proliferation of moyamoya type collateral arteries, greater on the left. Axial 3D-TOF MRA again shows the moyamoya collaterals in both basal ganglia (**f**)

Overall, 163 of 172 (95%) affected segments demonstrated negative remodeling. The mean remodeling index was 0.45 (SD 0.33) in affected distal ICA segments (n = 33) compared to 0.96 (SD 0.10) in unaffected distal ICA segments (n = 15). Complete obliteration of the vessel wall was most frequently seen in the proximal M1 segment (17/48, 35%), but was also seen in the distal ICA (9/48, 19%), mid M1 segment (13/48, 27%), distal M1 segment (11/48, 23%), proximal A1 segment (12/47, 26%), and distal A1 segment (4/47, 9%). Interobserver agreement of presence of negative remodeling within any given segment was good to very good (kappa = 0.78). An example is provided in Fig. 4.

### Vessel wall enhancement

Seventy-two of 172 (42%) affected segments enhanced (any degree or pattern). Twenty of 24 (88%) patients had at least one enhancing segment. The number of enhancing segments per patient ranged from 0 to 10 (median = 2). Among patients with enhancing segments, a combination of simultaneous enhancing and non-enhancing affected segments was present in 19/20 (95%). The likelihood of vessel wall enhancement varied by location of affected segment (Table 2). Enhancement was most common in the distal ICA segment and least frequent in the distal M1 and distal A1 segments.

**Table 2:** Prevalence of negative remodelling, wall thickening, and vessel wall enhancement amongst affected segments^a^

Segment	Negative remodeling	Wall thickening	Wall enhancement absent	Wall enhancement present^b^	Grade 2 wall enhancement
Distal ICA	34/36	17/36 (47%)	15/36 (42%)	21/36 (58%)	5/36 (14%)
Proximal M1	32/33 (97%)	13/33 (39%)	16/33 (45%)	15/33 (55%)	5/33 (15%)
Mid M1	27/28 (96%)	9/28 (32%)	16/28 (57%)	12/28 (43%)	3/28 (11%)
Distal M1	24/24 (100%)	5/24 (21%)	21/24 (88%)	3/24 (13%)	1/24 (4%)
Proximal A1	27/33 (82%)	11/33 (33%)	19/33 (58%)	14/33 (42%)	1/33 (3%)
Distal A1	17/20 (85%)	1/20 (5%)	17/20 (85%)	3/20 (15%)	0/20 (0%)

^a^Affected segments include those with any of the following: negative remodeling, wall thickening, and/or vessel wall enhancement

^b^Grade 1 or 2 vessel wall enhancement

Contrast enhancement was most frequently grade 1 (57/72, 79%) and less commonly grade 2 (15/72). Concentric contrast enhancement was present in 54/72 (75%) enhancing segments while eccentric contrast enhancement was present in 18/72 (25%) segments. Overall, there was a significant correlation between vessel wall enhancement and smaller lumen diameter (*p* < 0.001; OR 0.220, 95% CI 0.128–0.379) (see below for correlation to thickening). The number of segments with vessel wall enhancement was not correlated to disease duration (*p* = 0.922; OR 0.999, 95% CI 0.972–1.026) or Suzuki score (*p* = 0.477; 0.749, 95% CI 0.327–1.714). Interobserver agreement of both degree (grade, kappa = 0.72) and pattern (concentric or eccentric, kappa = 0.73) were both good.

### Vessel wall thickening and correlation to vessel wall enhancement and diameters

Vessel wall thickening was present in 82 of 172 (48%) affected segments, including areas of either eccentric (n = 20, 24%) or concentric (n = 62, 76%) involvement. Overall, the association of vessel wall thickening (any pattern) and contrast enhancement (any pattern or degree) was statistically significant (*p* < 0.001; OR 142.474. 95% CI 42.338–479.442) and 15/15 (100%) segments with grade II enhancement demonstrated vessel wall thickening.

The presence of concentric or eccentric wall thickening had a positive association with the degree of vessel wall enhancement (*p* < 0.001; OR 142.474, 95% CI 42.338–479.442). There was also a significant association between the pattern of vessel wall thickening and pattern of vessel wall enhancement (*p* < 0.001; OR 244.802; 95CI 50.461–1187.609), indicating concentric or eccentric thickening was associated with concentric or eccentric enhancement, respectively. There was a significant correlation between the presence of vessel wall enhancement and smaller outer wall diameter (negative remodeling) (*p* < 0.001; OR 0.535, 95CI 0.384–0.747). There was a significant association between vessel wall thickening and smaller vessel wall diameter (*p* = 0.004; OR 0.682, 95% CI 0.528–0.883), and between vessel wall thickening and smaller lumen diameter (stenosis) (*p* < 0.001; OR 0.345, 95CI 0.343–0.347). Interobserver agreement for presence of thickening within any segment was fair (kappa = 0.50).

## Discussion

The current study characterizes VWI findings of MMD in a predominantly adult North American population and reveals several important findings. Negative remodeling of affected segments is a near-constant feature with frequent areas of complete arterial obliteration, while patterns of vessel wall thickening or enhancement among affected segments are variable. Nearly all patients demonstrated vessel wall enhancement in at least one affected segment, though most affected segments did not enhance. Such enhancement was usually found in the proximal segments and rarely found in distal M1 or distal A1 segments. High grade vessel wall enhancement (eccentric or concentric) was present in some patients and always associated with vessel wall thickening. Vessel wall enhancement was not correlated to disease duration or stage. These findings have implications for diagnosis and, potentially, for use to assess disease activity, particularly in patients in this population.

The results indicate that radiologists should base a diagnosis of MMD primarily on the typical anatomic distribution of stenosis and negative remodeling of segments with VWI but can expect to see a wide variety of vessel wall findings within and between patients in terms of contrast enhancement and thickening. The findings suggest that recognition of concentric and eccentric enhancement as potential vessel wall features of MMD could help avert misdiagnosis of either vasculitis or atherosclerosis, respectively. This differentiation may be challenging since MMS due to either vasculitis or atherosclerosis is possible. The presence of simultaneous enhancing and non-enhancing segments without an area of positive outward remodeling may be useful, although further work directly comparing MMD to MMS would be useful to confirm these assertions. In some cases, additional correlation with atherosclerotic risk factors and/or CSF studies may still be needed.

A couple of prior studies have characterized VWI features of MMD at North American Centers. Mossa-Basha et al. found that use of multi-contrast VWI compared to luminal imaging alone improved differentiation of MMD (8 patients) compared to atherosclerotic MMS (n = 10) and vasculitic MMS (n = 3) in a cohort of non-Asian North American patients [7]. Furthermore, this study identified mild concentric CE only in 13% of affected segments. In distinction, we found that CE is more common and variable in pattern and degree. Similar to Mossa-Basha et al., we found that outward positive remodeling was absent in all cases of MMD. A study by Cogswell et al. assessed the distal ICA in 24 Patients with MMD on non-contrast VWI, reporting that luminal stenosis and negative remodeling was present in all affected distal ICA segments [8]. Our study similarly identifies negative remodeling and stenosis in affected ICA segments. In distinction to the current study, Cogswell et al. did not identify thickening of the involved vessel walls. It is possible that this discrepancy is accounted for by differences in methods; we utilized subjective visual assessment, whereas the prior study defined wall thickening as the difference in measurement between the lumen and outer wall diameters. With either method, assessment may be challenging due to the small size of the arterial walls, although visual assessment more likely reflects actual clinical practice. The current study builds upon these prior publications by further characterizing the findings by including a larger number of patients with MMD, a more granular analysis of arterial segments, and/or use of IV contrast.

Most prior work has assessed patient populations outside of North America, including South Korea [9–11], China [11, 12] Japan [13, 14], India [15], and Europe [16]. A key finding of the current study is near-ubiquitous presence of negative remodeling, which was also seen in studies from Asia and Japan [9–11, 13]. Although the full pathomechanism is still being explored, fibrosis and thinning of the tunica media is considered a central feature of stenotic vessels in MMD on available histopathology reports [17]. Ryoo et al. report that contrast enhancement was present in the distal ICA in most patients, including 73.5% on asymptomatic sides and 93.3% on symptomatic sides in a South Korean cohort [10]. In distinction, Kathuveetil et al. report segments with vessel wall enhancement in only 27% of patients in a cohort from India [15]. Differences in results may depend on numerous factors, such as the population studied, imaging and analysis methods, disease stage, and revascularization status. For example, the patients in the study by Ryoo et al. had a RI of 0.19 compared to 0.45 in the current study and about half of the patients studied by Kathuveetil et al. had prior revascularization [10, 15].

Some of this prior work indicates that high grade vessel wall enhancement may predict future disease progression or infarcts [14–16], although Ryoo found that vessel wall enhancement (not graded) was not associated with symptoms [10]. Roder et al. report a chronological occurrence of disease progression in the setting of high grade enhancement with subsequent decreased enhancement and disease stability [16]. Low grade enhancement was not associated with disease progression. While the current study does not assess the impact of vessel wall enhancement on disease progression, it adds to these prior studies by confirming that some degree of vessel wall enhancement can be seen in early or long-standing cases of MMD. The results also show that vessel wall enhancement was more common in segments that demonstrate increasing degrees of established stenosis and negative remodeling.

This study has some limitations. This is a retrospective analysis and patients were first imaged at various timepoints after initial clinical presentation. To maintain focus on first establishing expected VWI findings in this North American population, this study represents a single timepoint analysis and was not designed to assess the correlation of vessel wall findings to other cross-sectional or angiographic imaging findings or development of future stroke, symptoms, or disease progression. The study was performed at a single institution that predominantly manages adult patients with MMD, which could introduce referral bias. Characterization of findings in pediatric North American patients will require future work. Additionally, it is possible that results could vary somewhat with different VWI sequences across different institutions. Most vessel wall features, and the precise demarcation of segments were assessed qualitatively, and the precise results are subject to interobserver variability. Like most other intracranial VWI and MMD studies, pathologic correlation was not available. As mentioned, some of our findings, such as eccentric enhancement, differ from many prior reports and further studies to assess these findings are needed. Despite submillimeter resolution, the ability to characterize negatively remodeled vessels in MMD still has spatial limitations and likely contributed to the fair interobserver reliability for the assessment of vessel wall thickening. This study characterized MMD without comparison to a control group, precluding comparison of the rates of VWI findings to those of other diagnoses. By definition, MMD is categorized, in part, by the distribution of luminal narrowing and reporting distribution of findings may seem circular. However, the predominant areas of focus of this study were the features, prevalence, and distribution of vessel wall findings other than stenosis. Finally, the North American population is heterogenous in terms of both geographic location and ethnicity with the possibility of regional and ethnic differences.

## Conclusion

This study helps establish VWI findings in adult North American patients with MMD. Similar to the Asian population, negative remodeling was the most common finding. Most patients had both enhancing and non-enhancing abnormal segments. Vessel wall enhancement was most common in proximal segments, variable in terms of pattern or degree, and was correlated to the degree of stenosis and to smaller outer wall diameter.

## Data Availability

The datasets used and/or analyzed during the current study are available from the corresponding author on reasonable request.

## Abbreviations

VWI: Vessel wall imaging
MMD: Moyamoya disease
MMS: Moyamoya syndrome
TIA: Transient ischemic attack
ICA: Internal carotid artery
SD: Standard deviation
